# *In silico* identification of chilli genome encoded MicroRNAs targeting the 16S rRNA and *secA* genes of “*Candidatus* phytoplasma trifolii*”*

**DOI:** 10.3389/fbinf.2024.1493712

**Published:** 2025-01-06

**Authors:** Vineeta Pandey, Aarshi Srivastava, Ramwant Gupta, Haitham E. M. Zaki, Muhammad Shafiq Shahid, Rajarshi K. Gaur

**Affiliations:** ^1^ Department of Biotechnology, Deen Dayal Upadhyaya Gorakhpur University, Gorakhpur, Uttar Pradesh, India; ^2^ Department of Botany, Deen Dayal Upadhyaya Gorakhpur University, Gorakhpur, Uttar Pradesh, India; ^3^ Horticulture Department, Faculty of Agriculture, Minia University, El-Minia, Egypt; ^4^ Applied Biotechnology Department, University of Technology and Applied Sciences-Sur, Sur, Oman; ^5^ Department of Plant Sciences, College of Agricultural and Marine Sciences, Sultan Qaboos University, Al‐khod, Oman

**Keywords:** phytoplasma, ‘candidatus phytoplasma trifolii’, chilli, 16S rrna, SecA, miRNA

## Abstract

Phytoplasma, a potentially hazardous pathogen associated with witches’ broom, is an economically harmful disease-producing bacteria that damages chilli cultivation. Phytoplasma-infected plants display various symptoms that indicate significant disruptions in normal plant physiology and behaviour. Diseases caused by phytoplasma are widespread and have a major economic impact on crop quality and yield. This work focuses on identifying and examining chilli microRNAs (miRNAs) as potential targets against the 16S rRNA and *secA* gene of “*Candidatus* Phytoplasma trifolii” (“*Ca*. P. trifolii”) through plant miRNA prediction algorithms. Mature chilli miRNAs (CA-miRNAs) were collected and used to hybridise the 16S rRNA and *secA* genes. A total of four common CA-miRNAs were picked according to genetic consensus. Three algorithms applied in the present study suggested that the physiologically relevant, top-ranked miR169b_2 has a possibly specific site at nucleotide position 1,006 for targeting the ‘*Ca*. P. trifolii’ 16S rRNA gene. The circos algorithm was then utilised to create the miRNA-mRNA regulatory network. The free energy between the miRNA:mRNA duplex was also computed, and the best value of −17.46 kcal/mol was obtained for CA-miR166c_2. Currently, there are no suitable commercial ‘*Ca*. P. trifolii’-resistant chilli crops. As a result, the expected biological data provide useful evidence for developing ‘*Ca*. P. trifolii’-resistant chilli plants.

## Introduction

Chilli (*Capsicum annuum L.*) is a staple vegetable and spice crop, valued for its young green and red ripe fruits. As a medicinal plant, it is known to possess various pharmacologically and biochemically active compounds (Bosland, 1996; Powis et al., 2013). Chilli fruits are attributed to the richness and diversity of bioactive components, including capsaicinoids, carotenoids, and vitamins (Bal et al., 2019b; Bal et al., 2020a; Bal et al., 2020b). Consuming capsaicin in chilli has antioxidant properties and can bind and destroy cancer cells (Oh et al., 2010). Agriculture crops face numerous biotic and abiotic challenges, with phytoplasma-associated diseases being a major concern in many parts of the world. These diseases significantly reduce both production yield and quality. (Bertaccini et al., 2014). Phytoplasma, which causes little leaf disease, is one of the major constraints for chilli production and can result in significant economic losses (Singh and Singh, 2000). Phytoplasmas, which are prokaryotic wall-less bacteria that flourish in isotonic habitats in insect hemolymph and phloem tissues of plants. They possess a small genome, approximately 680–1,600 kb in size. Phytoplasmas are associated with over 600 diverse plant diseases worldwide (Bertaccini et al., 2014). Phloem-feeding insects, specifically leafhoppers and plant hoppers, serve as the principal vectors of phytoplasma transmission (Bertaccini et al., 2014). Phytoplasmas disease are associated with a variety of symptoms, including little leaves, virescence, large buds, shorter internodes, witches’ broom, massive calyx, phyllody, vascular discoloration, and floral abnormalities. The ability to classify phytoplasmas into groups and subgroups was made possible by the development of molecular techniques; this process mostly relied on the examination of the 16S rRNA gene sequence (Lee et al., 1998a; IRPCM, 2004). As the fundamental elements of the Sec translocation protein system, *secA*, *secE*, and *secY* have been found in onion yellow phytoplasma (OY) (Economou, 1999; Kakizawa et al., 2001). They are crucial for both protein movement and cell survival in *Escherichia coli*. Phytoplasma diseases have existed in India for over a century. Coconut root wilt disease was first observed in South Kerala in 1874 (Varghese, 1934), whereas first phytoplasma disease in chilli was reported in India by Singh and Singh (2000) and ‘*Candidatus* Phytoplasma trifolii’ causing witches broom disease in chillies was also reported by Rao et al. (2017). According to a recent study, the 16SrVI-D phytoplasma subgroup was associated with *Capsicum chinense* in India (Dutta et al., 2022).

MicroRNAs (miRNAs) are short (19–25 nucleotide) non-coding, single-stranded RNA molecules that exist naturally in plants and have evolved to be conserved (Finnegan and Matzke, 2003). In higher plants, the synthesis of miRNA gene (MIR) is controlled by RNA polymerase II. The miRNA gene is translated and generates single-standard polycistronic primary transcripts or primary miRNAs. These miRNAs regulate a wide range of biological activities in plants, including gene expression, differentiation, development, cell growth, and host-pathogen interactions (Millar, 2020; Islam et al., 2022). The post-transcriptional gene-silencing (PTGS) process known as miRNA-mediated RNA interference (RNAi) regulates or inhibits viral or non-viral infection by regulating host-virus interactions and providing antimicrobial innate immunity (Jin et al., 2022). Profiling miRNAs in mulberry phloem saps due to phytoplasma infection can help evaluate the molecular mechanisms underlying phytoplasma pathogenicity (Gai et al., 2018). The “*Ca*. P. trifolii”s’ gene were used as the target binding sites for chilli genome-encoded miRNAs, using a comprehensive multi-network strategy based on “*Ca*. P. trifolii” infection evaluation.

The major purpose of this study is to discover multiple host-derived miRNA binding sites in the 16S rRNA and *secA* genes that may be used to create transgenic chilli cultivars resistant to “*Ca*. P. trifolii”. This study used several miRNA prediction algorithms to detect microRNA-mRNA binding locations in the 16S rRNA and *secA* gene. These loci may be used to create hybrid/non-hybrid chilli plants resistant to “*Ca*. P. trifolii” and similar phytoplasma.

To get an in-depth comprehension of phytoplasma plant interactions during infection, it was also interesting to identify relevant targets for the most efficient CA-miRNAs. There have been no investigations on using amiRNA-based techniques to establish phytoplasma resistance in chilli plants, considering its potential for silencing “*Ca*. P. trifolii”. Further analysis of the anticipated locus-derived CA-miRNAs in the chilli genome was conducted to uncover new antiviral targets and comprehend the complicated relationships between the phytoplasma “*Ca*. P. trifolii” and the chilli host plants.

## Materials and methods

### *Capsicum annuum* CA-miRNA and target genome sequence (phytoplasma) retrieval

The miRNA sRNAanno database was used to retrieve 76 mature chilli microRNAs (CA-miRNAs) that have been experimentally confirmed with high confidence from chr1 to chr5 (Supplementary Table S1). The miRNA targets chosen for this analysis were phytoplasma 16S rRNA (Accession no. MZ557805) and *secA* (Accession no. MZ620707) gene sequences identified in our previous study of mixed infection in the chilli plant. The sequences were collected from the NCBI GenBank database (Supplementary Figure S1).

### Target prediction in 16S rRNA and *secA* of phytoplasma

Target prediction is a crucial factor in establishing reliable miRNA-mRNA interaction hybridization. Many target prediction algorithms have been used to identify the best miRNA target candidates. Each tool utilizes distinct criteria and methodologies to make predictions. We assessed five target prediction techniques documented in the literature to determine the most relevant CA-miRNAs for phytoplasma components silencing: RNAhybrid (Krüger and Rehmsmeier, 2006), TAPIR (Bonnet et al., 2010), RNA22 (MiRanda et al., 2006; Loher and Rigoutsos, 2012), MiRanda (Enright et al., 2003; John et al., 2004) and psRNATarget (Dai and Zhao, 2011; Dai et al., 2018). These tools calculate complementarity-based miRNA-mRNA binding. An effective computational method was employed to evaluate miRNA targets by examining three different prediction levels: individual, union and intersection (Supplementary Figure S1).

### Target prediction algorithms: RNAHybrid, tapirhybrid, RNA22, MiRanda and psRNATarget

A large number of plant miRNAs bind to their targets with perfect or almost perfect sequence complementarity (Llave et al., 2002; Reinhart et al., 2002). RNAHybrid, an online programme, allows users to identify miRNA targets using mRNA and miRNA minimum free energy (MFE) matching easily. We accepted the default parameters that were specified with hit per target of 1 with MFE threshold of −20 kcal/mol to get the more stable miRNA and mRNA heteroduplex. The Tapirhybrid method evaluates plant miRNAs in the target region for their seed-based interactions. With FASTA and RNAhybrid search capabilities, it is utilised to provide accurate miRNA target predictions, including target mimics. The free energy ratio of 0.2 and score of 9 were selected to increase the accuracy in the result (Table 1). Using RNA22, target locations with appropriate hetero-duplexes was predicted. Among the most delicate algorithmic components are non-seed interactions, pattern detection, MFE, and site compatibility (MiRanda et al., 2006). The study was conducted with sensitivity and specificity of 63% and 61% respectively, the GU region allowed in seed region with no limit and MFE for heteroduplex was −12 kcal/mol for identifying more than 60% accurate and consistent interactions. MiRanda is the most extensively used standard computational approach for predicting miRNA targets (Table 1). The MiRanda method was executed using free energy of −15 kcal/mol and score threshold of 140 led to better alignment and sustained interactions (Table 1). The psRNATarget algorithm, finds that the target phytoplasma components mRNA region and CA-miRNAs are reversely complementary (Dai et al., 2018). Target-site accessibility was evaluated by calculating the unpaired energy (UPE) using the psRNATarget approach. The interaction between miRNA and mRNA was computed using user-specified factors and an expected value cut-off of 5 (Table 1) determining the most probable binding locations while reducing the risk of false positives.

**TABLE 1:** The distinguishing features of the five target prediction tools.

	Algorithms	Seed pairing	Target site accessibility	Translation inhibition	Source	Parameter used
RNAhybrid	Interamolecular hybridization	+	+	+	http://bibiserv.techfak.unibielefeld.de/rnahybrid (accessed on 30 March 2024)	Hit per target = 1 MFE = −20 kcal/mol
Tapirhybrid	FASTA	+	+	−	http://bioinformatics.psb.ugent.be/webtools/tapir (accessed on 25 April 2024)	Free energy ratio = 0.2 Score = 9
RNA22	FASTA	−	+	−	https://cm.jefferson.edu/rna22/Interactive/(accessed on 20 May 2024)	Sensitivity = 63%, Specificity = 61% GU region allowed in seed region = no limit MFE for heterduplex = −12 kcal/mol
miRanda	Local alignment	+	+	+	http://www.microrna.org/ (accessed on 31 May 2024)	Free energy = −15 kcal/mol Score threshold = 140Gap Extend penalty = −4.00 Gap Open penalty = −9.00
psRNATarget	Smith-Waterman	−	+	+	https://www.zhaolab.org/psRNATarget/analysis?function = 2 (accessed on 1 June 2024)	Expectation score = 5, HSP size = 19 Penalty for G:U pair = 0.5 Penalty for opening gap = 2

### CA-miRNA–16S rRNA and *secA* interaction mapping

The Circos method was used in the R programme to construct an interaction map between 16S rRNA, *secA*, and CA-miRNAs (Krzywinski et al., 2009) (Supplementary Figure S1) to enable the detection and study of similarities and differences resulting from miRNA and mRNA interaction**.** Circos method allows for effective visualisation of sequence alignments, genome mapping, hybridisation arrays, and genotyping experiments (Krzywinski et al., 2009).

### Thermodynamic stability: free energy (ΔG) evaluation of duplex binding

Sequence alignment is beneficial in predicting miRNA-mRNA interactions, but the thermodynamic aspects of miRNA-mRNA complexes provide critical information for determining hybridization durability (Riolo et al., 2020). Most miRNA-targeting prediction approaches use the free energy (ΔG) of the expected interaction to assess the thermodynamic characteristics of the miRNA-mRNA complex. RNAcofold, an online tool (http://rna.tbi.univie.ac.at/cgi-bin/RNAWebSuite/RNAcofold.cgi), predicts the duplex (miRNA and mRNA) free energy (ΔG) of interactions (Bernhart et al., 2006). Using the miRNA-target pair from psRNATarget, the necessary 16S rRNA and *secA* sequences, as well as CA-miRNAs, were studied with the RNAcofold default parameters.

## Result

### CA-miRNA target prediction on phytoplasma

**FIGURE 1:**
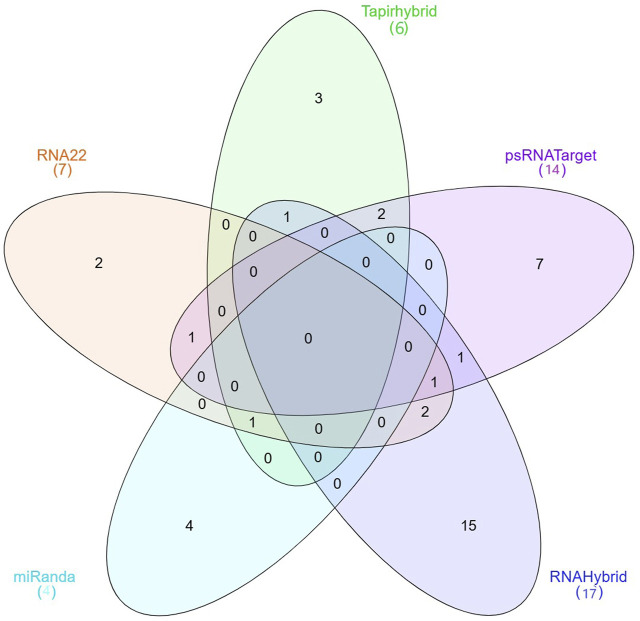
The Venn diagram plot of chilli-encoded miRNAs has been created for all five methods. Chilli-encoded miRNAs target 48 locations on “*Ca.*P.trifolii” 16S rRNA and *secA*. Furthermore, the computational tools used in this work confirm the total number of targeting sites for 33 CA-miRNAs that interact with 16S rRNA and *secA*. Three mathematical approaches (Tapirhybrid, RNA22, and miRanda) predicted the presence of a single CA-miRNA (CA_miR169b_2).

miRNAs with a precise or near-perfect match to their target mRNAs regulate post-transcriptional gene expression through mechanisms such as translation inhibition and cleavage. The microRNA causes mRNA cleavage and subsequent degradation by binding with complementarity in the seed region and base pairing in the central section (Pasquinelli, 2012). This degradation, which is sequence-specific, relies on RNA hydrolysis, leading to effective silence (Dykxhoorn et al., 2003). Limited compatibility, on the other hand, typically results in lower gene expression because it prevents the host from translating the targeted mRNA (Bonnet et al., 2004). This study revealed host miRNAs capable of selectively targeting known phytoplasma 16S rRNA and *secA* isolates in chilli plants. Because miRNA binding to target RNA genomes is quite diverse, we employed five algorithmic approaches (RNAHybrid, Tapirhybrid, RNA22, MiRanda and psRNATarget) to determine the binding strength and phytoplasma relevance of the 76 known CA-miRNAs (Supplementary Table S1). When numerous *in silico* approaches were employed to establish target alignment with phytoplasma 16S rRNA and *secA* phytoplasma components, around 48 target transcripts were identified to be targeted by these 76 known CA-miRNAs (Figure 1). Out of the 76 known miRNAs, three algorithms identified one CA-miRNA (i.e., CA_miR169b_2) (Table 2). RNAHybrid predicted seventeen miRNA targets. Similarly, Tapirhybrid identified six miRNAs that target 16S rRNA. Both RNAHybrid and Tapirhybrid revealed no miRNA with the binding affinity to the *secA* gene. Furthermore, four miRNAs in RNA22 showed an interaction for their target, each having one target within the 16S rRNA, whereas *secA* had three target sites (Table 2). Similarly, MiRanda confirmed that both 16S rRNA and *secA* were targeted by four distinct miRNAs (Table 2). While evaluating the psRNATarget data, we observed ten and four high-probability miRNA binding sites for 16S rRNA and *secA*, respectively, whereas CA-miR5300_2 targets two different locations in 16S rRNA.

**TABLE 2:** List of chilli known miRNA showing target within “*Candidatus* phytoplasma trifolii” 16S rRNA and secA through a different algorithm.

Known chilli miRNA	Algorithms predicted miRNA within 16S rRNA and *secA*				
	Tapirhybrid	RNA22	psRNATarget	RNAHybrid	miRanda
CA-miR169b_2	16S rRNA	16S rRNA	----------	----------	16S rRNA
CA-miR319c_2	----------	*secA*	16S rRNA	16S rRNA	----------
CA-miR399e_2	----------	16S rRNA, *secA*	----------	16S rRNA	----------
CA-miR482a_1	----------	*secA*	----------	----------	----------
CA-miR482a_2	----------	16S rRNA	----------	----------	----------
CA-miR1446a_2	----------	----------	16S rRNA	----------	----------
CA-miR156b_2	----------	----------	16S rRNA	16S rRNA	----------
CA-miR159a_1	----------	----------	*secA*	----------	----------
CA-miR159b_1	----------	----------	*secA*	----------	----------
CA-miR159c_1	----------	----------	*secA*	----------	----------
CA-miR160_1	----------	----------	----------	16S rRNA	----------
CA-miR160_2	----------	----------	16S rRNA	16S rRNA	----------
CA-miR166c_2	16S rRNA	----------	16S rRNA	----------	----------
CA-miR166d_2	----------	----------	----------	16S rRNA	----------
CA-miR168a_1	16S rRNA	----------	16S rRNA	----------	----------
CA-miR168a_2	----------	----------	----------	16S rRNA	----------
CA-miR168b_1	----------	----------	----------	16S rRNA	----------
CA-miR168b_2	----------	----------	16S rRNA	----------	----------
CA-miR169a_1	----------	----------	----------	16S rRNA	----------
CA-miR169a_2	----------	----------	----------	16S rRNA	----------
CA-miR169b_1	----------	----------	----------	16S rRNA	----------
CA-miR171a_2	16S rRNA	----------	----------	----------	----------
CA-miR171b_2	16S rRNA	----------	----------	----------	----------
CA-miR172b_2	----------	----------	----------	----------	*secA*
CA-miR319c_1	----------	----------	----------	16S rRNA	----------
CA-miR399e_1	----------	----------	----------	16S rRNA	----------
CA-miR399g_1	----------	----------	----------	16S rRNA	----------
CA-miR399g_2	----------	----------	----------	16S rRNA	----------
CA-miR403a_1	----------	----------	----------	----------	*secA*
CA-miR403a_2	----------	----------	----------	----------	16S rRNA
CA-miR482a_2	----------	----------	----------	16S rRNA	----------
CA-miR5300_1	----------	----------	----------	16S rRNA	----------
CA-miR5300_2	16S rRNA	----------	16S rRNA (at two different locus)	----------	----------
CA-miR6026_1	----------	16S rRNA	16S rRNA, *secA*	----------	----------

### Chilli-miRNA target prediction at 16S rRNA

**FIGURE 2:**
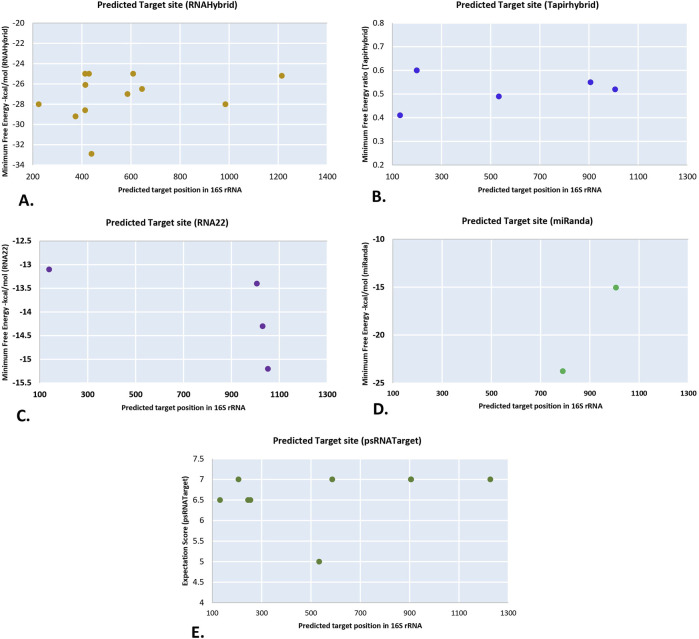
The “five algorithms” strategy predicted unique chilli CA-miRNAs and their high-confidence binding regions across *Ca.P.trifolii’s* 16S rRNA. **(A)** CA-miRNA binding sites were identified using RNAhybrid. **(B)** Tapirhybrid reported the target’s CA-miRNA positions and MFE ratio. **(C)** RNA22 predicts miRNA binding affinity sites. **(D)** miRanda reported the target’s CA-miRNA spots. **(E)** psRNATarget indicates CA-miRNA binding sites.

**FIGURE 3:**
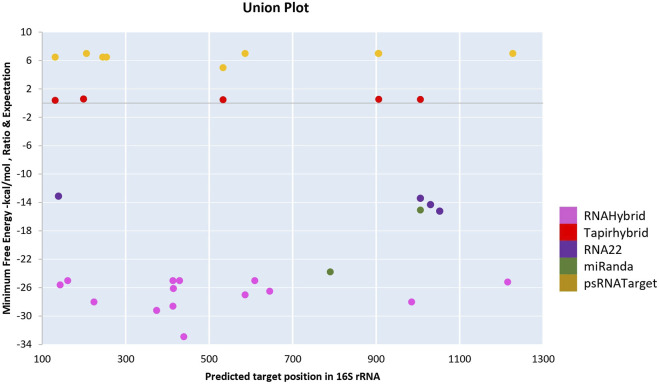
The union plot depicts every predicted binding region identified by each method used. Coloured dots represent numerous copies of the binding spots for miRNA targets by different computational methods.

This analysis found that thirty-four of the seventy-six known CA-miRNA transcripts encoded by chr1 to chr5 had targets in ‘*Ca*. P. trifolii’ 16S rRNA gene. RNAHybrid showed a total of seventeen miRNA transcripts targeting the 16S rRNA, with CA-miR399 transcripts indicating four targets (Figure 2A). Likewise, tapirhybrid predicted six targeting sites, including two transcripts of CA-miR171 (Figure 2B). In RNA22, four separate miRNA members (i.e., CA-miR6026_1, CA-miR399e_2, CA-miR169b_2, and CA_miR482a_2) targeted the four different prediction sites (Figure 2C). CA-miR403a_2 and CA-miR169b_2 showed cleavage affinity for 16S rRNA in miRanda (Figure 2D). psRNATarget identified ten targeting sites for nine miRNA transcript each targeting one sites except CA-miR5300_2 which individually targeted at two different loci in 16S rRNA (Figure 2E) (Figure 3). MiRanda confirmed CA-miR403a_1 and CA-miR172b_2 as targeting two distinct loci (Figure 3).

### CA-miRNAs targeting *secA*

**FIGURE 4:**
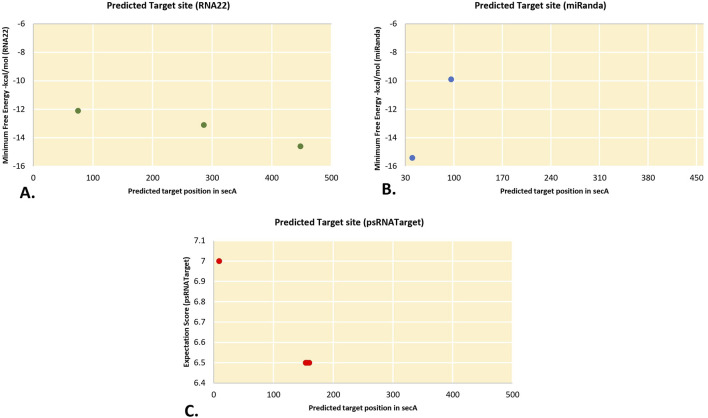
The “three algorithms” strategy predicted unique chilli CA-miRNAs and their high-confidence binding regions across ‘*Ca.*P.trifolii’ *secA*. **(A)** RNA22 predicts miRNA binding affinity sites. **(B)** miRanda reported the target’s CA-miRNA sites. **(C)** psRNATarget indicates CA-miRNA binding sites along with expectation scores.

Bacterial *Sec* protein transfer involves the *secA* protein. The translocation of proteins across the cell membrane that is dependent on ATP is mediated by it. According to Xue et al. (2023) *secA* most likely aids in the survival of phytoplasmas by moving proteins across the cell membrane. We obtained data for the *secA* gene from three target prediction algorithms. MiRanda, RNA22, and psRNATarget each predicted two, three, and four *secA* target sites, respectively (Figures 4A–C). In psRNATarget, transcripts of CA-miR159 targeted three of the targeting sites. However, RNA22 predicted three different binding sites by CA_miR319c_2, CA_miR399e_2, and CA_miR482a_1.

### Consensual identification of CA-miRNAs

**FIGURE 5:**
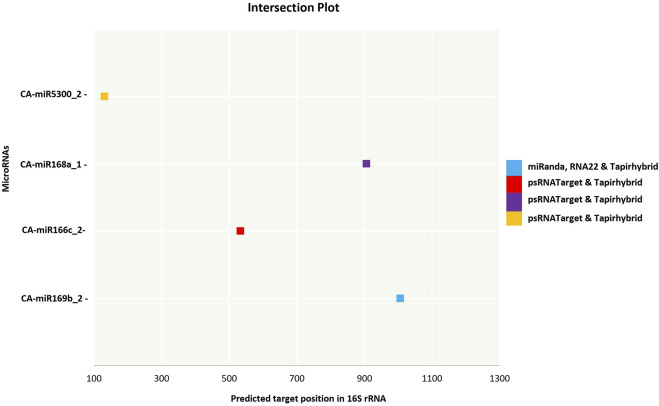
An intersection graph illustrates the most prevalent CA-miRNAs found using at least twodistinct approaches at homologous loci. Colour codes are provided in the figure showing algorithms.The expected cut-off score (psRNATarget), the minimum free energy (MFE) (RNAhybrid, miRanda, and RNA22), and the MFE ratio (Taphirhybrid) are presented.

The current study was carried out primarily on the consensus of the target binding loci of CA-miRNAs obtained through multiple approaches. We chose four CA-miRNAs, CA-miR169b_2, CA-miR166c_2, CA-miR168a_1, and CA-miR5300_2, considering consensus nucleotide spots 1,006, 533, 906, and 131, respectively (Tables 3) (Figure 5). Only one CA-miRNA, miR169b_2, was identified by combining nucleotide consensus sites at location 1,006 using three approaches (RNA22, TapirHybrid, and MiRanda).

**TABLE 3:** High-confidence binding sites for consensus CA-miRNAs that target the 16S rRNA gene of “*Candidatus* phytoplasma trifolii” that were predicted using several computational approaches.

Known chilli miRNA	Position RNAhybrid	Position TAPIR	Position RNA22	Position miRanda	Position psRNATarget	MFE* RNAhybrid	MFE ratio TAPIR	MFE** RNA22	MFE* miRanda	Expectation psRNATarget
CA-miR169b_2	----------	1,006	1,006	1,006	----------	----------	0.52	−13.4	−15.05	----------
CA-miR166c_2	----------	533	----------	----------	533	----------	0.49	----------	----------	5
CA-miR168a_1	----------	906	----------	----------	906	----------	0.55	----------	----------	7
CA-miR5300_2	----------	131	----------	----------	131	----------	0.41	----------	----------	6.5

*MFE: minimum free energy (Kcal/mol) **MFE: maximum folding energy of heteroduplex (Kcal/mol).

### Mapping of miRNA- “*Candidatus* phytoplasma trifolii” 16S rRNA and *secA* gene interaction

**FIGURE 6:**
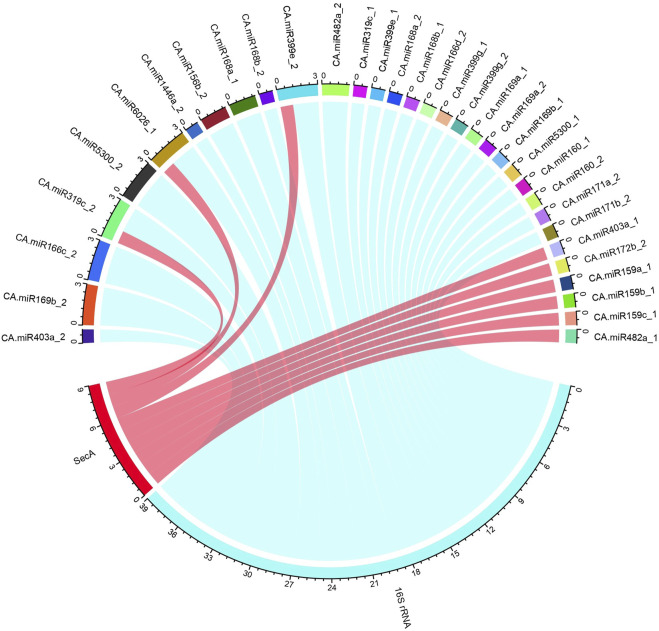
The “*Ca.*P.trifolii” 16S rRNA and *secA* gene schematic representation for the chilli-target interaction. The known CA-miRNAs retrieved from sRNAanno dataset and their targets against the 16S rRNA and *secA* gene are summarized in a circular plot (Circos) constructed with the R-program. The outer ring represents the genetic components of “*Ca*. P. trifolii” and known CA-miRNAs. The coloured lines reflect the interaction of both 16S rRNA and *secA* with the target.

To correctly integrate biologically valid data for investigating the miRNA-host gene, we utilised the R-tool to create circos plots for miRNA targets (Table 2). To enable best visualisation and readability, this mapping between the CA-miRNAs with their 16S rRNA and *secA* gene targets were done (Figure 6).

### Thermodynamic stability: free energy (ΔG) estimation for miRNA–mRNA heterodimer

The free energy (ΔG) of miRNA-mRNA duplex for those miRNAs that were supported by at least two predicted tools were evaluated. The miRNA-mRNA complex is thought to be highly thermodynamically stable, with as stronger miRNA-mRNA association when the ΔG of the complex is low (i.e., greater negative ΔG) which strengthens the miRNA’s regulatory influence on the target mRNA (Bernhart et al., 2006). This constitutes essential information because it increases the likelihood that stable miRNA-mRNA binding will be recognized as an actual interaction (Riolo et al., 2020). The RNAcofold algorithm’s free energy (ΔG) estimation was based on the alignment (miRNA-mRNA) result of psRNATarget. Four duplexes were identified, with the lowest free energy (ΔG) of > −15 kcal/mol for CA-miR166c_2, CA-miR166c_2, CA-miR168a_1 and CA-miR168b_2 for 16S rRNA (Table 4). CA-miR6026_1 had the lowest binding energy for *secA*, which was −12.34 kcal/mol.

**TABLE 4:** Duplex free energy (ΔG) of top four known CA-miRNAs, including binding range, length of target, with miRNA alignment:Target duplex.

Known chilli miRNA	miRNA_length	Target_start	Target_end	miRNA_aligned_fragment	Alignment	Target_aligned_fragment	ΔG (Kcal/mol) heterodimer binding
CA-miR166c_2	21	533	553	GGAAUGUUGUUUGGCUCGAGG	:::::::.:..:::::	GAUAGAGGCAAGCGGAAUUCC	−17.46
CA-miR319c_2	22	245	266	AGAGCUUUCUUCAGUCCACACA	::::.::.::::::	UUUCGGCAAUGGAGGAAACUCU	−10.67
CA-miR5300_2	22	81	102	UGGUAUGCUUUGGUUGGGAAAG	:.:.::::.:.::.:.	ACCUUCUUACGAAGGUAUGCUU	−10.43
CA-miR5300_2	22	131	152	UGGUAUGCUUUGGUUGGGAAAG	.::.:..::::::::::	UUGUUAGAGUAAAAGCCUACCA	−10.58
CA-miR6026_1	22	254	275	UUCUUGGCUAGAGUUGUGUUGC	.:.::::::.::.:::	UGGAGGAAACUCUGACCGAGCA	−4.20
CA-miR1446a_2	21	206	226	CUUUGGGGGUUUGAGUUCAGA	..::::::.::.:.:	ACACGGCCCAAACUCCUACGG	−12.68
CA-miR156b_2	22	586	608	GCUCUCUAUGCUUC-GGUCAUCA	::::::::::.:::. .::	GGAACACCAGAAGCGUAGGCGGC	−13.23
CA-miR166c_2	21	1,227	1,246	GGAAUGUUGUUUGGCUCGAGG	:::.:..::.::.:::.	UGUCGGGGUGAAUA-CGUUCU	−16.00
CA-miR168a_1	21	906	926	CCCGCCUUGCAUCAACUGAAU	:::::::.::::::.:	AUACAGGUGGUGCAUGGUUGU	−16.59
CA-miR168b_2	22	905	926	CCUGCCUUGCAUCAACUGAAUU	.:::::::.::::::.:	GAUACAGGUGGUGCAUGGUUGU	−16.18
CA-miR6026_1	22	9	30	UUCUUGGCUAGAGUUGUGUUGC	.:.:::::.:::::	UUUAAUUAUUUCUAGUCAAAAA	−12.34
CA-miR159a_1	21	154	174	UUUGGAUUGAAGGGAGCUCUA	::.:…:::::..:::	GAAACUUUUUUUCAAAUUAAA	−7.00
CA-miR159b_1	21	154	174	UUUGGAUUGAAGGGAGCUCUA	::.:…:::::..:::	GAAACUUUUUUUCAAAUUAAA	−7.00
CA-miR159c_1	21	154	174	UUUGGAUUGAAGGGAGCUCUA	::.:…:::::..:::	GAAACUUUUUUUCAAAUUAAA	−7.00

### Known CA-miRNAs secondary structures

**FIGURE 7:**
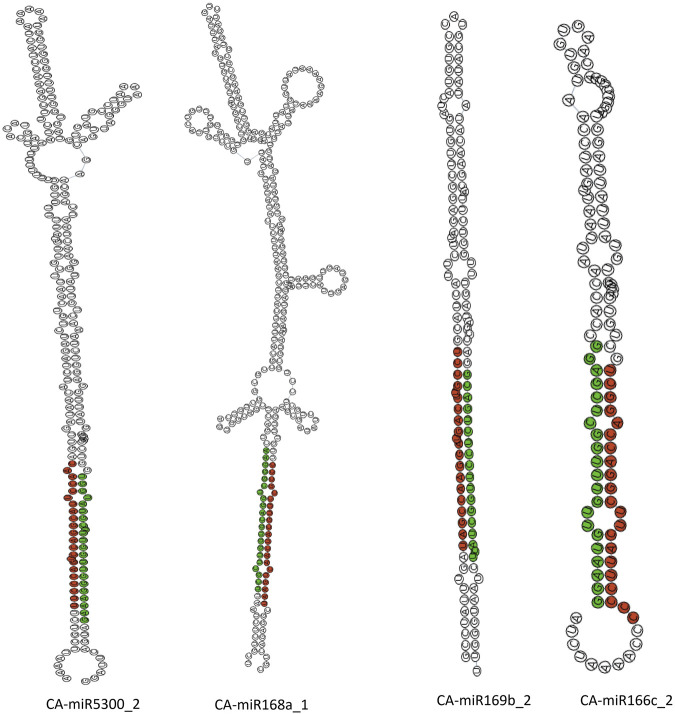
Secondary structure of known CA-miRNAs: those with a greater abundance are coloured red, whereas those with a low abundance are coloured green.

**FIGURE 8:**
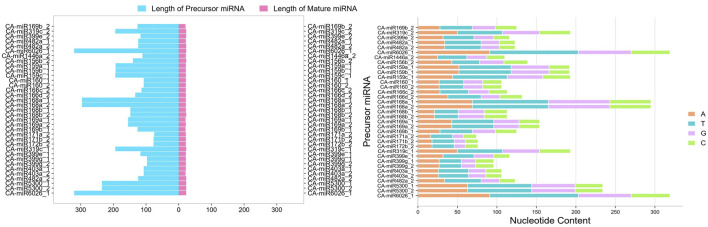
Mature miRNA length and precursor miRNA length were measured. Pink indicates mature miRNAs, whereas blue indicates the length of miRNA precursors. Nucleotide composition of the precursor miRNA was also determined.

The sRNAanno database (Chen et al., 2021) was used to predict stable secondary structures for known CA-miRNAs (Figure 7). Precursors for mature CA-miRNAs were retrieved from same database. The secondary structures of four pre-miRNA precursors as predicted by the intersection of two consensus algorithms at the same locus were identified. We identified the important attributes of thirty-three precursor miRNAs that showed targets for either 16S rRNA or *secA*, including MFE, Adjusted Minimum Folding Free Energy (AMFE), Minimum Folding free Energy Index (MFEI), length precursor, length of mature miRNA, nucleotide and GC content (Figure 8). The MFE is the most important determinant for assessing precursors’ stable secondary structures. According to Bonnet et al. (2004), precursor microRNAs must have less folding energy compared to different non-coding RNAs. The RNAfold tool were used to accessed the MFE value of precursor miRNA (Lorenz et al., 2011). These known CA-miRNAs precursors were found to have lowered MFE values (range from −27.00 to −134.20 kcal/mol) (Table 5). In this work, the precursor length ranged from 116–319 nucleotides (Figure 8), and the (G + C) % varied from 34.9% to 54.8%. The AMFE measured between −26.54 and −49.52 kcal/mol, with an MFEI of −0.58 to −1.24 kcal/mol. Using standard characteristics, the topmost stable secondary structure of precursor was CA-miR6026_1 (MFE: 134.20 kcal/mol, MFEI: 1.16 kcal/mol).

**TABLE 5:** The characteristics of the known precursors of chilli were identified.

Known chilli miRNA (Acronyms)	MFE^a^ (kcal/mol)	AMFE^b^	MFEI^c^	(G + C) %
CA-miR169b_2	−61.90	−49.52	−1.11	44.8
CA-miR319c_2	−94.10	−48.75	−1.10	44.5
CA-miR399e_2	−55.10	−47.5	−1.23	38.7
CA-miR482a_1	−50.50	−41.05	−1.18	34.9
CA-miR482a_2	−50.50	−41.05	−1.18	34.9
CA-miR1446a_2	−59.50	−54.09	−1.24	43.6
CA-miR156b_2	−49.80	−35.82	−0.82	43.8
CA-miR159a_1	−76.20	−39.68	−1.03	38.5
CA-miR159b_1	−76.20	−39.68	−1.03	38.5
CA-miR159c_1	−88.80	−46.01	−1.11	41.4
CA-miR160_1	−53.10	−50.09	−1.11	45.2
CA-miR160_2	−53.10	−50.09	−1.11	45.2
CA-miR166c_2	−30.00	−26.54	−0.61	43.3
CA-miR166d_2	−46.50	−35.22	−0.90	39.3
CA-miR168a_1	−99.30	−33.66	−0.77	44.0
CA-miR168a_2	−99.30	−33.66	−0.77	44.0
CA-miR168b_1	−47.10	−32.04	−0.58	54.8
CA-miR168b_2	−47.10	−32.04	−0.58	54.8
CA-miR169a_1	−54.50	−35.38	−0.94	37.7
CA-miR169a_2	−54.50	−35.38	−0.94	37.7
CA-miR169b_1	−61.90	−49.52	−1.11	44.8
CA-miR171a_2	−27.20	−36.75	−0.91	40.5
CA-miR171b_2	−27.00	−35.52	−0.90	39.4
CA-miR172b_2	−45.60	−35.07	−0.35	53.3
CA-miR319c_1	−94.10	−48.75	−1.10	44.5
CA-miR399e_1	−55.10	−47.5	−1.22	38.8
CA-miR399g_1	−45.70	−47.60	−1.11	42.7
CA-miR399g_2	−45.70	−47.60	−1.11	42.7
CA-miR403a_1	−50.70	−47.83	−1.21	39.6
CA-miR403a_2	−50.70	−47.83	−1.21	39.6
CA-miR482a_2	−50.50	−41.05	−1.18	34.9
CA-miR5300_1	−80.30	−34.31	−0.89	38.5
CA-miR5300_2	−80.30	−34.31	−0.89	38.5
CA-miR6026_1	−134.20	−42.06	−1.16	36.4

^a^ MFE: minimum free energy.

^b^ AMFE: adjusted minimum free energy.

^c^ MFEI: minimum free energy index.

## Discussion

Chilli fruit and its supplementary components have significant applications and a diverse range of bioactive chemicals in farming, nourishment, pharmaceuticals, healthcare, and skincare sector. Its by-products are also useful in the field of textile (Havsteen, 2002; Dixon and Pasinetti, 2010; Liu et al., 2013). Aside from its restricted genetic base, chilli revenue is severely affected due to its susceptibility against to biotic and abiotic pressures. Phytoplasmas are non-culturable prokaryotic bacteria responsible for a variety of plant diseases and are spread by insect’s feed on phloem. Chilli is prone to a variety of diseases, among which little leaf disease, caused by phytoplasmas, responsible for major economic losses (Singh and Singh, 2000).

In eukaryotes, microRNAs (miRNAs) are well-conserved, short endogenous non-coding RNAs that use sequence complementarity to target and destroy mRNA. In plant miRNAs often exhibit perfect base-pairing with target sites whereas animal miRNAs establish imperfect duplexes with target sequences, hence confounding the prediction of direct targets (Pasquinelli, 2012). MiRNAs typically suppress target expression in plants and animals by causing mRNA de-adenylation and degradation, as well as limiting translation (Pasquinelli, 2012). Research has explored complex host-virus interactions and employed computational approaches to study miRNAs targeting plant viruses (Akhter and Khan, 2013; Ashraf et al. 2022; 2023; Iqbal et al., 2017; Jabbar et al., 2019; Shahid et al., 2022). In our earlier study, we predicted and examined the mature locus-derived microRNAs in the chilli and papaya genome that were expected to be chilli leaf curl virus (ChiLCV) and papaya leaf curl virus (PaLCuV) targets based on *in silico* criteria (Pandey et al., 2024; Srivastava et al., 2024).

In this *in silico* research, we attempted for the first time to align mature chilli CA-miRNAs with the genomic sequence of the 16S rRNA and *secA* gene of ‘*Ca*. P. trifolii’ targets in order to identify miRNA-mRNA binding loci hypothesised for comprehending complex host-phytoplasma interactions. The survival of phytoplasma relies on its two primary components, 16S rRNA and *sec (A, Y,* and *E)* genes. The 3′end of 16S rRNA interacts with proteins S1 and S21, which are believed to be associated with protein synthesis beginning (Czernilofsky et al., 1975). The 16S rRNA gene is frequently used in phylogenetic investigations (Weisburg et al., 1991) because it is primarily conserved across diverse bacteria and archaea (Coenye and Vandamme, 2003). Similarly, proteins released via the *Sec* system are anticipated to be crucial throughout the infection process as they facilitate protein translocation. So, this work employs “*Ca*. P. trifolii” 16S rRNA and *secA* as CA-miRNA targets, which might be useful for similar phytoplasma sequences.

We investigated the effectiveness of computational strategies for assessing miRNA target prediction data to filter out false-positive outcomes. We developed a reliable method for validating these predictions at the individual, union, and intersection stages. Algorithmic prediction provides quick ways for identifying putative host-derived target regions for miRNA in phytoplasma genomes. The parameters vary depending on the algorithm or tool and may be adjusted to fine-tune the settings or increase the degree of sensitivity for expected spots. Five different approaches were utilised for target prediction: RNAHybrid, Tarpirhybrid, RNA22, miRanda, and psRNATarget. We applied all five approaches to determining the MFE and target inhibition sites.

Two or more algorithms may jointly identify a number of putative CA-miRNAs targets and miRNA-mRNA duplexes (Figure 3). Target gene destruction is induced by plant miRNAs by the application of perfect or near-perfect complementary base pairing (Jones-Rhoades et al., 2006). The present study shows that a collection of consensus CA-miRNAs may target ‘*Ca*. P. trifolii’ genomic components (16S rRNA and *secA* gene). Furthermore, three algorithms identified CA-miR169b_2 as targeting 16S rRNA at the same consensus hybridisation site (i.e., 1,006), and because this specific miRNA’s target region was proven by three approaches, more research could be undertaken on it (Figure 5). miR169 is largely conserved across plant species and may be activated by drought and salt environments in rice (Sunkar and Jagadeeswaran, 2008). Free energy estimation is a dynamic characteristic of miRNA and target binding. Previous research has identified a strong link between free energy and both translational repression and seed hybridization binding (Doench and Sharp, 2004). The thermodynamic stability of the miRNA-mRNA heterodimer was assessed using free energy analysis to track site availability for secondary structure duplex identification (Peterson et al., 2014). To validate miRNA-mRNA interactions, we calculated the free energy of the heterodimer (Table 4). Our prediction indicates that the chilli-encoded miRNA-phytoplasma-mRNA duplex is highly stable at low free energy levels (Table 4). The increased stability of the RNA duplex is attributed to the stronger interaction between the miRNA and mRNA (Lewis et al., 2005; Huang et al., 2010).

We applied union and intersection methods to decrease false positive predictions. When it comes to detecting genuine and false targets, union techniques rely on merging many target prediction tools. An intersecting method is fundamentally different, relying on the integration of two or more computational algorithms to increase the specificity of anticipated targets by reducing insensitivity (Witkos et al., 2011). Our target prediction outcomes showed that both computational methods performed optimally when identifying and estimating the optimum targets (Figures 3, 5). Based on the manner of miRNA-target identification, MFE is another significant component that influences miRNA-target interaction during result validation (Pinzón et al., 2017). Setting a lower MFE value increases the possibility of miRNA-target building complexes (Kertesz et al., 2007). For miRanda analysis, a strict cut-off value of −15 kcal/mol was used to filter out miRNA candidates. Similarly, to confirm host-phytoplasma interaction, RNA hybrid analysis was performed with an MFE cut-off value of −20 kcal/mol present investigation, we identified 17 candidate miRNA hybridization binding sites with low MFEs and free energy for duplex formation (Enright et al., 2003). Although MFE plays an important role in the formation of miRNA-mRNA duplexes, it fails to guarantee that interactions result in functional alterations. In the present investigation, we identified 14 candidate miRNA hybridization binding sites with low MFEs and free energy values for duplex formation by using psRNATarget and RNAcofold.

These candidate CA-miRNAs have potential transgenic targets for the 16S rRNA and *secA* genomes, as well as a greater possibility of forming miRNA-phytoplasma mRNA complexes. We selected best four experimentally confirmed CA-miRNAs with identified high-confidence targets from ‘*Ca*. P. trifolii’ (Table 3) (i.e., CA-miR169b_2, CA-miR166c_2, CA-miR168a_1 and CA-miR5300_2) and predict their secondary structure through sRNAanno database. The amiRNA-based silencing technique has been effectively proven in numerous agricultural plants for controlling emerging plant pathogens (Niu et al., 2006; Ali et al., 2013; Petchthai et al., 2018).

To the best of our knowledge, this is the first-time known CA-miRNAs have targeted at phytoplasmic components. Our computational study of “*Ca*. P. trifolii” gene silencing may provide a novel strategy for the creation of anti-phytoplasma agents. Furthermore, we developed a technique for minimising the new anti-phytoplasma impacts of host-derived miRNAs on “*Ca*. P. trifolii”. The *in silico* research aimed to provide a basis for experimental validation to determine whether known CA-miRNAs could confer resistance to “*Ca*. P. trifolii” in plants. The expression of CA-miR169b_2 in transgenic chilli varieties to silence “*Ca*. P. trifolii” target genes might help us gain insight into crucial host-virus interactions.

## Conclusion

In India, phytoplasma has emerged as a major agricultural threat, affecting a wide range of crops and “*Ca*. P. trifolii” lowers the quantitative production of chilli cultivars. In this study, we used computational techniques to predict and thoroughly investigate possible miRNA from chilli against “*Ca*. P. trifolii” 16S rRNA and *secA* gene. The best CA-miRNA for interacting with the “*Ca*. P. trifolii” was discovered to be miR169b_2. Our findings suggest that miR169b_2 may be a viable and successful treatment strategy for “*Ca*. P. trifolii” infection in chilli cultivars. Large-scale transgenic chilli cultivar development must be substantiated by pathological implications. As a result, the next challenge will be to find the crucial miR169b_2 targets involved in silencing the “*Ca*. P. trifolii” genome’s 16S rRNA gene, as well as determining their involvement in a genome-editing-based conversion system. Using chilli transformation procedures, predicted new targets can be created to create “*Ca*. P. trifolii” -resistant chilli cultivars. Chilli transformation processes can be used to generate expected new objectives for “*Ca*. P. trifolii” -resistant chilli cultivars.

## Data Availability

The original contributions presented in the study are included in the article/Supplementary Material, further inquiries can be directed to the corresponding authors.

## Supplementary material

SUPPLEMENTARY FIGURE S1 Flow chart showing all computational tools used in this research.

SUPPLEMENTARY TABLE S1 List of 76 mature known CA-miRNAs retrieved from sRNAanno database, along with its location on chromosomes.
