# Supplementary material for: In silico identification of chilli genome encoded MicroRNAs targeting the 16S rRNA and secA genes of “Candidatus phytoplasma trifolii”
Source: Front Bioinform. 2025 Jan 6;4:1493712. doi: 10.3389/fbinf.2024.1493712 (PMC11743513; doi:10.3389/fbinf.2024.1493712)
Supplement: Supplementary file 1 [file Table1.docx]

| **Chromosome**  **Supplementary Table 1** List of 76 mature known CA-miRNAs retrieved from sRNAanno database, along with its location on chromosomes. | **Known miRNA in Chilli** | **Acronyms** | **Start** | **Stop** | **Strand** | **Mature Sequence** |
| --- | --- | --- | --- | --- | --- | --- |
| **chr01** | miR166b | CA-miR166b_1 | 493291 | 493311 | - | 5':UCGGACCAGGCUUCAUUCCCC:3' |
|  | miR166b | CA-miR166b_2 | 493376 | 493396 | - | 5':GGAAUGUUGUCUGGCUCGAGG:3' |
|  | miR319c | CA-miR319c_1 | 2960346 | 2960367 | - | 5':CUUGGACUGAAGGGAGCUCCCU:3' |
|  | miR319c | CA-miR319c_2 | 2960496 | 2960517 | - | 5':AGAGCUUUCUUCAGUCCACACA:3' |
|  | miR159a | CA-miR159a_1 | 3711594 | 3711614 | + | 5':UUUGGAUUGAAGGGAGCUCUA:3' |
|  | miR159a | CA-miR159a_2 | 3711445 | 3711465 | + | 5':GAGCUCUCUGAAGUCCAAAAG:3' |
|  | miR159b | CA-miR159b_1 | 3712942 | 3712962 | + | 5':UUUGGAUUGAAGGGAGCUCUA:3' |
|  | miR159b | CA-miR159b_2 | 3712793 | 3712813 | + | 5':GAGCUCUCUGAAGUCCAAAAG:3' |
|  | miR399e | CA-miR399e_1 | 7292837 | 7292857 | + | 5':UGCCAAAGGAGAGUUGCCCUG:3' |
|  | miR399e | CA-miR399e_2 | 7292763 | 7292784 | + | 5':GGGCAGCUCUCCGUUUGGCAGA:3' |
|  | miR171a | CA-miR171a_1 | 27166116 | 27166136 | - | 5':UAUUGGCCUGGUUCACUCAGA:3' |
|  | miR171a | CA-miR171a_2 | 27166081 | 27166101 | - | 5':UGAUUGAGCCGUGUCAAUAUC:3' |
|  | miR168a | CA-miR168a_1 | 46651903 | 46651923 | - | 5':CCCGCCUUGCAUCAACUGAAU:3' |
|  | miR168a | CA-miR168a_2 | 46652156 | 46652176 | - | 5':UCGCUUGGUGCAGGUCGGGAA:3' |
|  | miR6026 | CA-miR6026_1 | 69102857 | 69102878 | - | 5':UUCUUGGCUAGAGUUGUGUUGC:3' |
|  | miR6026 | CA-miR6026_2 | 69103130 | 69103151 | - | 5':AACGCAACUAUAGCCAAGAGAA:3' |
|  | miR403a | CA-miR403a_1 | 208787463 | 208787483 | - | 5':UUAGAUUCACGCACAAACUCG:3' |
|  | miR403a | CA-miR403a_2 | 208787528 | 208787547 | - | 5':CGUUUGUGCGUGAAUCUGAC:3' |
|  | miR168b | CA-miR168b_1 | 303173464 | 303173484 | + | 5':UCGCUUGGUGCAGGUCGGGAC:3' |
|  | miR168b | CA-miR168b_2 | 303173568 | 303173589 | + | 5':CCUGCCUUGCAUCAACUGAAUU:3' |
|  | miR166c | CA-miR166c_1 | 306510797 | 306510817.00 | + | 5':UCGGACCAGGCUUCAUUCCCC:3' |
|  | miR166c | CA-miR166c_2 | 306510715 | 306510735 | + | 5':GGAAUGUUGUUUGGCUCGAGG:3' |
| **chr02** | miR171b | CA-miR171b_1 | 4461881 | 4461901 | - | 5':UAUUGGCCUGGUUCACUCAGA:3' |
|  | miR171b | CA-miR171b_2 | 4461844 | 4461864 | - | 5':UGAUUGAGCCGUGUCAAUAUC:3' |
|  | miR171d | CA-miR171d_1 | 152119381 | 152119401 | - | 5':UAUUGGCCUGGUUCACUCAGA:3' |
|  | miR171d | CA-miR171d_2 | 152119301 | 152119321 | - | 5':UGAUUGAGCCGUGCCAAUAUC:3' |
| **chr03** | miR166d | CA-miR166d_1 | 20683156 | 20683176 | + | 5':UCGGACCAGGCUUCAUUCCCC:3' |
|  | miR166d | CA-miR166d_2 | 20683067 | 20683087 | + | 5':AGAAUGUUGUCUGGUUCGCAA:3' |
|  | miR167a | CA-miR167a_1 | 21431704 | 21431724 | - | 5':UGAAGCUGCCAGCAUGAUCUA:3' |
|  | miR167a | CA-miR167a_2 | 21431652 | 21431671 | - | 5':GAUCAUAUGGUAGCUUCACC:3' |
|  | miR167c | CA-miR167c_1 | 145850525.00 | 145850545 | + | 5':UGAAGCUGCCAGCAUGAUCUA:3' |
|  | miR167c | CA-miR167c_2 | 145850579 | 145850598 | + | 5':GAUCAUGUGGCAGCAUCACC:3' |
|  | miR399g | CA-miR399g_1 | 219314108 | 219314128 | + | 5':GGGCGACUCUCUAUUGGCAUG:3' |
|  | miR399g | CA-miR399g_2 | 219314162 | 219314182 | + | 5':CGCCAAAGGAGAGCUGCCCUA:3' |
|  | miR479 | CA-miR479_1 | 249358836 | 249358856 | - | 5':UGAGCCGAACCAAUAUCACUC:3' |
|  | miR479 | CA-miR479_2 | 249358883 | 249358903 | - | 5':CGUGAUAUUGGUUCGGCUCAC:3' |
|  | miR166e | CA-miR166e_1 | 249936988 | 249937008 | + | 5':UCGGACCAGGCUUCAUUCCCC:3' |
|  | miR166e | CA-miR166e_2 | 249936878 | 249936898 | + | 5':GGAAUGUUGUCUGGCUCGAAG:3' |
|  | miR169a | CA-miR169a_1 | 256615477 | 256615496 | + | 5':UAGCCAAGGAUGACUUGCCU:3' |
|  | miR169a | CA-miR169a_2 | 256615589 | 256615609 | + | 5':AGGCGUCGUCUGAGGCUAGUC:3' |
|  | miR171e | CA-miR171e_1 | 258571304 | 258571324 | - | 5':UGUUGGAACGGCUCAAUCAAA:3' |
|  | miR171e | CA-miR171e_2 | 258571249 | 258571269 | - | 5':UGAUUGAGCCGUGCCAAUAUC:3' |
|  | miR162a | CA-miR162a_1 | 267603605 | 267603625 | + | 5':UCGAUAAACCUCUGCAUCCAG:3' |
|  | miR162a | CA-miR162a_2 | 267603545 | 267603565 | + | 5':GGAGGCAGCGGUUCAUCGGUC:3' |
|  | miR162b | CA-miR162b_1 | 267691909 | 267691929 | + | 5':UCGAUAAACCUCUGCAUCCAG:3' |
|  | miR162b | CA-miR162b_2 | 267691843 | 267691863 | + | 5':GGAGGCAGCGGUUGAUCGAUC:3' |
|  | miR162c | CA-miR162c_1 | 268472913 | 268472933 | - | 5':UCGAUAAACCUCUGCAUCCAG:3' |
|  | miR162c | CA-miR162c_2 | 268472979 | 268472999 | - | 5':GGAGGCAGCGGUUGAUCGAUC:3' |
|  | miR162d | CA-miR162d_1 | 268521005 | 268521025 | - | 5':UCGAUAAACCUCUGCAUCCGG:3' |
|  | miR162d | CA-miR162d_2 | 268521071 | 268521091 | - | 5':GGAGGCAGCGGUUGAUCGAUC:3' |
|  | miR162e | CA-miR162e_1 | 268574550 | 268574570 | - | 5':UCGAUAAACCUCUGCAUCCAG:3' |
|  | miR162e | CA-miR162e_2 | 268574610 | 268574630 | - | 5':GGAGGCAGCGGUUCAUCGAUC:3' |
|  | miR1446a | CA-miR1446a_1 | 276000707 | 276000728 | - | 5':UGAACUCUCUCCCUCAAUGGCU:3' |
|  | miR1446a | CA-miR1446a_2 | 276000640 | 276000660 | - | 5':CUUUGGGGGUUUGAGUUCAGA:3' |
|  | miR156b | CA-miR156b_1 | 277524882 | 277524902 | + | 5':UUGACAGAAGAUAGAGAGCAC:3' |
|  | miR156b | CA-miR156b_2 | 277524978 | 277524999 | + | 5':GCUCUCUAUGCUUCGGUCAUCA:3' |
|  | miR159c | CA-miR159c_1 | 277676140 | 277676160 | + | 5':UUUGGAUUGAAGGGAGCUCUA:3' |
|  | miR159c | CA-miR159c_2 | 277675989 | 277676009 | + | 5':GAGCUCCUUGAAGUCCAAACG:3' |
| **chr04** | miR169b | CA-miR169b_1 | 23668078 | 23668097 | - | 5':UAGCCAAGGAUGACUUGCCU:3' |
|  | miR169b | CA-miR169b_2 | 23667993 | 23668010 | - | 5': UAGCCAAGGAUGACUUGCCU:3' |
|  | miR172a | CA-miR172a_1 | 27359077 | 27359097 | - | 5':AGAAUCUUGAUGAUGCUGCAU:3' |
|  | miR172a | CA-miR172a_2 | 27359162 | 27359182 | - | 5':GUAGCAUAAUCAAGAUUCACA:3' |
|  | miR482a | CA-miR482a_1 | 183086746 | 183086766 | - | 5':UGUGGGUGGGGUGGAAAGAUU:3' |
|  | miR482a | CA-miR482a_2 | 183086665 | 183086686 | - | 5':UCUUUCCUACUCCUCCCAUACC:3' |
|  | miR482b | CA-miR482b_1 | 187884907 | 187884928 | - | 5':UUUCCAAUUCCACCCAUUCCUA:3' |
|  | miR482b | CA-miR482b_2 | 187884959 | 187884979 | - | 5':AAUUGGUGGGUUGGAAAGCUU:3' |
| **chr05** | miR172b | CA-miR172b_1 | 7683415 | 7683435 | + | 5':AGAAUCUUGAUGAUGCUGCAG:3' |
|  | miR172b | CA-miR172b_2 | 7683337 | 7683357 | + | 5':GCAGCAUCUUCAAGAUUCACA:3' |
|  | miR5300 | CA-miR5300_1 | 59799241 | 59799262 | - | 5':UUCCCAGUCCAGGCAUUCCAAC:3' |
|  | miR5300 | CA-miR5300_2 | 59799050 | 59799071 | - | 5':UGGUAUGCUUUGGUUGGGAAAG:3' |
|  | miR172c | CA-miR172c_1 | 227093193 | 227093213 | - | 5':GGAAUCUUGAUGAUGCUGCAG:3' |
|  | miR172c | CA-miR172c_2 | 227093266 | 227093286 | - | 5':GGAGCAUCAUCAAGAUUCACA:3' |
|  | miR172d | CA-miR172d_1 | 237652837 | 237652857 | - | 5':AGAAUCUUGAUGCUGCUGCAU:3' |
|  | miR172d | CA-miR172d_2 | 237652907 | 237652927 | - | 5':GCAGCAUUAUCAAGAUUCACA:3' |
|  | miR160 | CA-miR160_1 | 238245135 | 238245155 | - | 5':GCGUAUGAGGAGCCAAGCAUA:3' |
|  | miR160 | CA-miR160_2 | 238245199 | 238245219 | - | 5':UGCCUGGCUCCCUGUAUGCCA:3' |
