# Supplementary figures and images for: In silico identification of chilli genome encoded MicroRNAs targeting the 16S rRNA and secA genes of “Candidatus phytoplasma trifolii”
Source: Front Bioinform. 2025 Jan 6;4:1493712. doi: 10.3389/fbinf.2024.1493712 (PMC11743513; doi:10.3389/fbinf.2024.1493712)

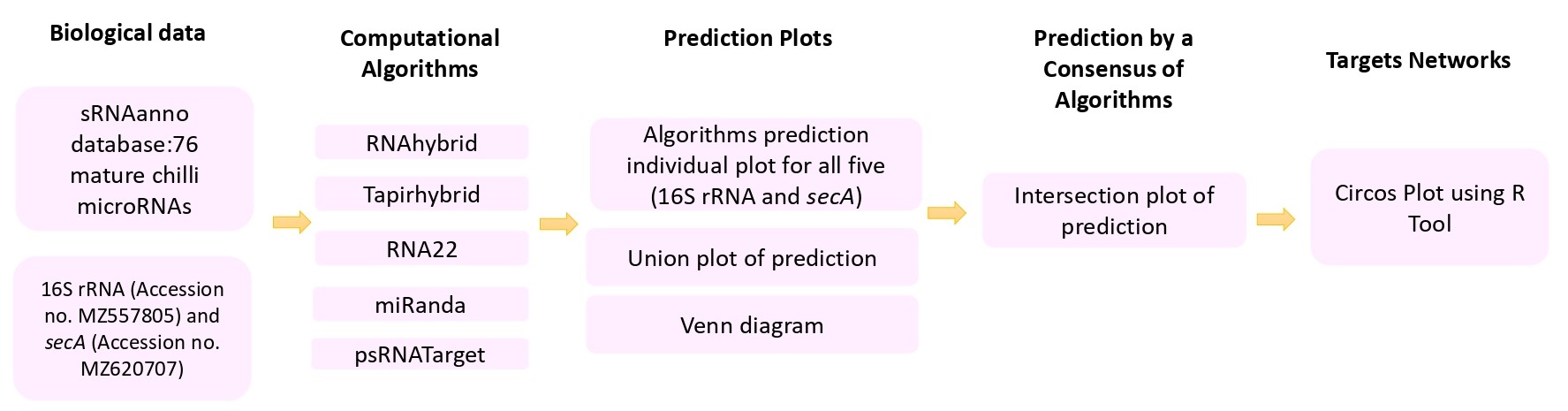

Supplement: Supplementary file 2 [file Image1.jpeg]
